# An efficient method for developing SNP markers based on EST data combined with high resolution melting (HRM) analysis

**DOI:** 10.1186/1756-0500-3-51

**Published:** 2010-03-02

**Authors:** Tokuko Ujino-Ihara, Yuriko Taguchi, Yoshinari Moriguchi, Yoshihiko Tsumura

**Affiliations:** 1 Department of Forest Genetics, Forestry and Forest Products Research Institute, Tsukuba, Ibaraki, 305-8687, Japan

## Abstract

**Background:**

In order to identify single nucleotide polymorphisms (SNPs) efficiently from a species with a large genome, SNPs were identified from an expressed sequence tag (EST) database combined with High Resolution Melting (HRM) analysis.

**Findings:**

A total of 574 sequence tagged sites (STSs) were generated from *Cryptomeria japonica *and HRM analysis was used to screen for polymorphisms in these STS markers. STSs were designed in two ways: 1) putative SNP sites were identified by comparing ESTs from specific contigs, then 226 primer pairs designed for the purpose to amplify these SNPs; 2) 348 primer pairs were randomly designed using reads from the 3' end of cDNA. HRM analysis revealed that 325 markers among eight individuals were polymorphic, and that STSs, including putative SNP sites, exhibited higher levels of polymorphism.

**Conclusion:**

Our results indicate that the combination of SNP screening from an EST database combined with HRM analysis is a highly efficient way to develop SNP markers for expressed genes. This method will contribute to both genetic mapping and the identification of SNPs in non-model organisms.

## Background

Sugi, *Cryptomeria japonica*, is one of the most important commercial tree species in Japan. Recently, linkage maps have been constructed for this species, based on co-dominant markers including RFLP (Restriction Fragment Length Polymorphism), CAPS (Cleaved Amplified Polymorphic Sequences), and SSR (Simple Sequence Repeat) markers [1-3]. Although these linkage maps include hundreds of markers, more DNA markers are required to generate a denser linkage map. Furthermore, some markers do not segregate in all crosses, thus increasing the number of markers can greatly enhance the scope and resolution of QTL analyses using the progenies of controlled crosses.

*C. japonica *has large genome (~10100 Mbp) [4], therefore full genomic sequences have not been obtained. On the other hand, because of the importance of this species to Japanese forestry, expressed sequence tags (ESTs) have been generated using several types of tissues from several individuals [5-7]. Redundant sequences found in EST databases can be a useful resource for mining SNPs or developing DNA markers, since mapping expressed genes to a linkage map makes the map more useful for QTL analysis or Marker-Assisted Selection (MAS). The discovery of SNPs in ESTs has been conducted for several species using programs such as PolyBayes [8] and QualitySNP [9]. We used QualitySNP in our study because it allowed us to consider both paralogous genes and sequence errors without having a sequence quality file and reference sequences.

After mining putative SNPs from a database, methods to identify STS polymorphisms are required. HRM analysis is an efficient SNP detection method that identifies differences in the shapes of melting curves between different genotypes using an intercalating dye that binds to the double-stranded DNA [10]. In short, the melting curve changes its shape due to mismatches in heteroduplexes or variation of nucleotides in homoduplexes that exist in the post-PCR mixture. HRM analysis has considerable advantages over other SNP detection methods because it only requires PCR products stained with specific dyes. As a result of these advantages, HRM analysis has gained popularity and applied to not only mutation screening of specific genes but genetic mapping in plant species [11-13]. Using HRM, SNPs have been developed in almond based on alignments of peach/almond ESTs obtained from public database [14]. In this study, we report an efficient way to develop a large number of HRM markers for genetic mapping in non-model organisms by combining SNP mining from an EST database with HRM analysis, using publicly available software.

## Methods

### Primer design

**Figure 1:**
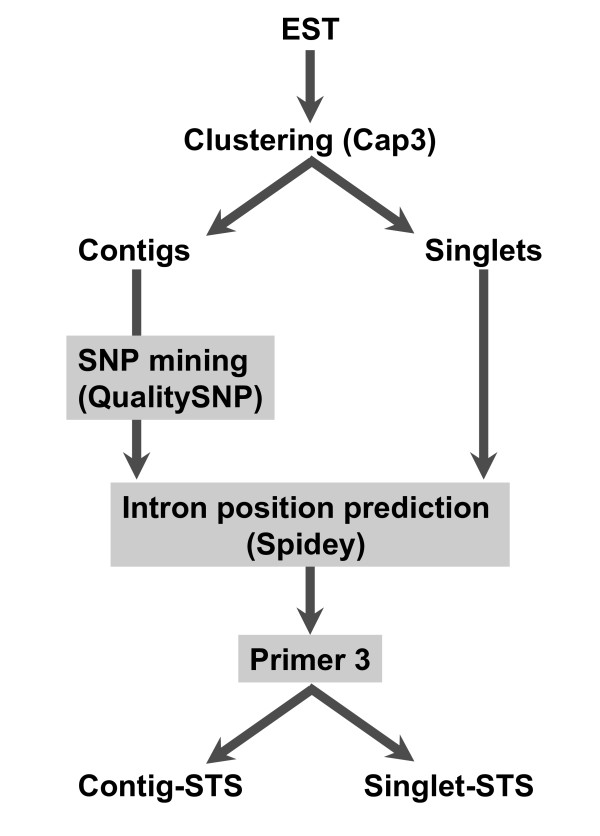
**Diagrammatic representation of primer design method**.

Primer design method is illustrated in Figure 1. EST sequences of *C. japonica *were obtained from the NCBI EST database. Putative contaminant DNA (from bacterial genomic DNA, cpDNA, and mtDNA) was excluded on the basis of the results of BLAST analysis. Clusters of ESTs were then identified by the CAP3 program; the minimum length and percentage identity of overlap was set to 100 and 95 respectively [15]. For the obtained contigs, possible SNPs were screened using QualitySNP with the default settings, except for the threshold of over-all similarity between haplotypes, which was set to 0.9. The primers were designed for the predicted putative SNP target sites. Hereafter, these STSs are referred to as 'contig-STSs'. The primers were also randomly designed from readings from the 3' end of cDNA (3'-ESTs) among the resultant singlet sequences, and these were designated 'singlet-STSs'. In both cases, possible exon-intron boundaries were screened by the Spidey program [16], by comparing the cDNA of *C. japonica *to genomic sequences of *Arabidopsis *with option for interspecies alignment. Although *C. japonica *is phylogenetically distant from *Arabidopsis*, it was reported that 11.5% of ESTs derived from 5'-terminal sequences of cDNAs exhibited significant similarities to gene coding sequence of *Arabidopsis *at nucleotide sequence level [7]. Any detected exon-intron boundaries were avoided in designing primers. All primer pairs were designed using Primer 3 [17] to amplify amplicons within the range 90 bp-120 bp. The result obtained by Spidey and QualitySNP were incorporated into input files for primer3 using perl scripts developed in-house.

### Plant material

Polymorphisms of the STS markers were screened with eight *C. japonica *individuals. Six of the individuals were from natural populations, representing the species' natural distribution in Japan: Yakushima, Shimowada, Ishinomaki, Oki, Ajigasawa, and Ashu. Two further sources of parental material were used for mapping populations, YI38 and YI96. These two samples were derived from a local cultivar on Kyushu-island. Since SNP markers detected by QualitySNP may include spurious markers (because paralogous sequences may be assembled in a contig), but those that segregate in an expected manner are likely to be true SNPs, the validity of the HRM markers was tested by segregation analysis using the progeny of YI38 and YI96. DNA of all plant material was extracted using a modified CTAB method [18].

### High Resolution Melting Analysis

PCR amplification was performed in 10 μl reaction volumes containing 200 μM of each dNTP, 0.8 units of *GXL *DNA polymerase (Takara Co. Ltd.), 1 × buffer provided for the *GXL *DNA polymerase that contained 1.0 mM MgCl_2_, 0.2 μM of each primer, 0.5 ul DMSO, 0.5 ul of EvaGreen, and 5 ng of template DNA (Fukuoka et al., personal communication). The PCR amplification was carried out for 10 min at 95°C, followed by 45 cycles of 40 sec at 94°C, 30 sec at 60°C, and 15 sec at 72°C. High resolution Melting Analysis was carried out using a Lightcycler 480 (Roche) according to the manufacturer's instructions. If the amplification product yield was low, we decreased the PCR annealing temperature to 56°C.

## Results and Discussion

### SNP discovery and the development of STS markers

A total of 55634 ESTs of *Cryptomeria japonica *were available on the NCBI EST database. After removing possible organelle and bacterial genes, 55530 ESTs were assembled into 10368 contigs and 13783 singlets. In the QualitySNP analysis, contigs with at least four ESTs were considered for SNP screening. Of the 10368 contigs, 3809 (3310919 bp) were screened and 1246 SNPs with high confidence were found in 314 contigs (8.2% of the total number of contigs screened). The overall SNP frequency was one SNP per 2657.2 bp for the 3809 screened contigs.

In the case of maritime pine [19], SNPs were also surveyed from EST data using Phred [20,21], Phrap http://www.phrap.org, and PolyBayes. SNP abundance was estimated as one SNP per 660 bp in a set of 940 contigs representing 942216 bp. A similar study was also carried out for white spruce, and 12264 SNPs were found when 6459 contigs of at least two cDNA clones were surveyed with Polybayes [22]. These authors estimated an overall frequency of one SNP per 700 nucleotide sites. Compared to previous studies, the proportion of contigs with a putative SNP site and the SNP frequency in *C. japonica *appears to be much lower. This may be the result of the different software used, the numbers of ESTs screened, or the number of individuals (or varieties) examined in the EST database. In our study, parameters used in EST clustering and SNP detection were severe to avoid assembling paralogous sequences into contigs; this may have also affected the SNP discovery rate.

**Figure 2:**
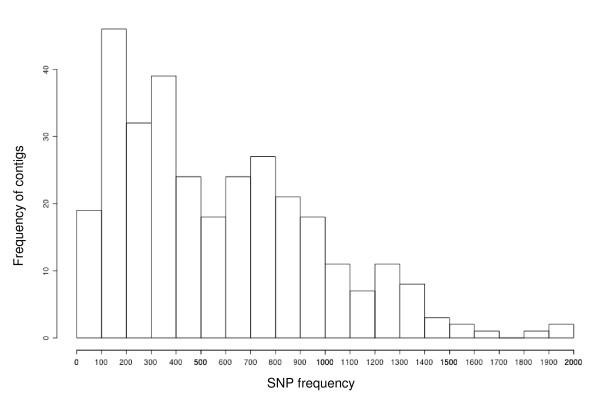
**The distribution of SNP frequency of 314 contig-STSs**. The y-axis shows the frequency of contigs for which the SNP frequency is given on the x-axis.

The predicted SNP distribution was uneven and there was high variation in SNP frequency among the 314 contigs, ranging from one SNP every 3.6 bp to 1967 bp (Figure 2). In a study of the DNA variation in *C. japonica*, the frequency of segregation sites occurring in transcribed regions ranged from one segregation site every 89.79 bp to 1047 bp [23]. SNP frequency was also estimated in maritime pine and one SNP was found every 102.6 bp [19]. Considering these SNP frequencies, D-values from QualitySNP (which indicate the probability of paralogous genes being present in a cluster), and polynucleotide sequences (which often cause repeat number errors at the beginning or end of a read), 55 contigs were excluded from primer design after manual inspection of the assemblage. Consequently, a total of 259 primer pairs were designed from the 314 putative SNPs (contig-STS).

For comparison, 412 primer pairs were also randomly designed from 3' ESTs (singlet-STS). These primer pairs were designed from 3' ESTs because previous studies have suggested that STSs derived from 3' ESTs are more polymorphic than STSs derived from 5' ESTs [24].

As a consequence, 226 and 348 STSs were successfully amplified from contig-STS and singlet-STS, respectively (Table 1). Single fragments were obtained for most primer pairs because the amplicons were designed to amplify short fragments (90-120 bp); it was also an advantage of HRM markers.

**Table 1:** Summary of the development of HRM markers

Source	Total STS No. tested	Total STS No. amplified	Polymorphic STS
Contigs	260	226	173 (76.5%)
Singlets	412	348	153 (43.9%)

### Detection of nucleotide differences for STSs by HRM analysis

**Figure 3:**
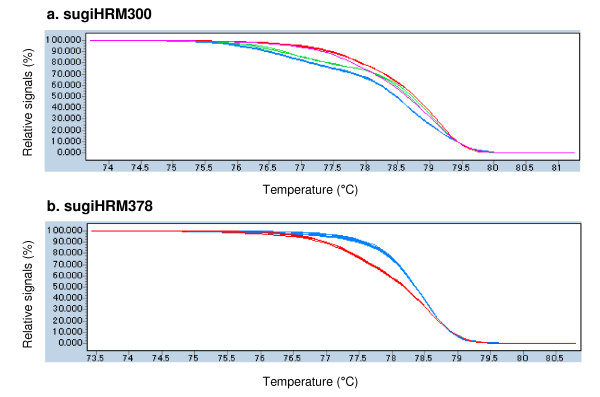
**Normalized and shifted high resolution melting (HRM) curve profiles of two HRM amplicons**. HRM profiles of eight *C. japonica *individuals described in Materials and Methods were shown. a. sugiHRM300 showing four different profiles, b. sugiHRM378 showing two different profiles. Both amplicons included SNP site predicted by QualitySNP from EST data (G/A for sugiHRM300 and T/A for sugiHRM378). Genotypes for each profile were not indicated because they were not validated by sequencing. Both HRM markers segregated in expected manner in F_2_ progeny of YI38 and YI96.

Obtained STSs were then analyzed by HRM analysis. One-hundred and seventy-three of the 226 STSs from the contig-STSs (76.5%) were polymorphic among the eight individuals used in this study (Figure 3); this was a much higher efficiency than obtained by randomly designed primers from singlet-STSs, as 43.9% of them were polymorphic (Table 1). Segregation of 148 STSs that segregated between YI38 and YI96 were also tested using F_2 _progeny of them. The results revealed that 128 of the 148 STSs (86.5%) exhibited the expected segregation from parental genotypes (data not shown), thus detected polymorphisms are unlikely to have resulted from nucleotide differences in paralogous genes. Higher polymorphism in the contig-STSs and the segregation in expected manner of those HRM markers indicated that QualitySNP detected actual SNPs in the transcribed region.

When SNPs were screened by CAPS analysis, 72.3% of STSs were polymorphic [2]. The detection efficiency of CAPS is comparable to that of HRM analysis of contig-STSs, however 15 individuals were screened using 24 or 36 endonucleases in the CAPS analysis. The screening of CAPS markers takes more time and labor. In the SSCP analysis, 37.3% of tested STSs were polymorphic among 10 individuals with 12 different electrophoresis conditions [24]. Therefore, if the SNP frequency was similar in both cases, HRM analysis is likely to be more sensitive than SSCP analysis. As described above, HRM analysis shows higher sensitivity even when they were analyzed under one experimental condition. Therefore HRM analysis is a more efficient way to detect SNPs in terms of the sensitivity, cost, time and labor in genomic mapping of non-model organisms. Changing experimental condition will further improve the detection efficiency. It is important to note that the panel individuals and individuals used in the cDNA library construction were not same. The absence of polymorphism in the HRM analysis was sometimes unrelated to the sensitivity of the technique, but due to the absence of the predicted SNPs in panel DNA.

## Conclusions

We demonstrated here that using HRM analysis for converting expressed sequences to DNA markers is very useful for genetic mapping. Although there are SNP typing methods that have higher throughput than HRM analysis, HRM analysis can be alternative method for moderate scale genome project in aspects of time and cost. The method described here used mainly publicly available resources, therefore it is easily applicable to non-model organisms with large genome size, such as *C. japonica*.

## Competing interests

The authors declare that they have no competing interests.

## Authors' contributions

TUI designed the strategy for SNP discovery and HRM analysis, and developed the perl scripts needed in primer design. YT conducted the experiments for HRM analysis. YM conducted the segregation analysis for mapping populations. YT supervised the project. TUI wrote the paper. All authors read and approved the final manuscript.
